# Cognition and Indicators of Dietary Habits in Older Adults from Southern Brazil

**DOI:** 10.1371/journal.pone.0147820

**Published:** 2016-02-19

**Authors:** Vivian Francielle França, Aline Rodrigues Barbosa, Eleonora D’Orsi

**Affiliations:** 1 Doutoranda, Universidade Federal de Santa Catarina, Programa de Pós Graduação em Nutrição, Florianópolis, Brasil; 2 Doutora, Departamento de Nutrição, Centro de Desportos, Universidade Federal de Santa Catarina, Programa de Pós Graduação em Nutrição, Florianópolis, Brasil; 3 Doutora, Departamento de Saúde Pública, Universidade Federal de Santa Catarina, Programa de Pós Graduação em Saúde Coletiva, Florianópolis, Brasil; Nathan Kline Institute and New York University School of Medicine, UNITED STATES

## Abstract

**Objective:**

To assess the association between unhealthy dietary habits and cognition in older adults from Southern Brazil.

**Methods:**

This cross-sectional study analyzed data from the second wave of a population- and household-based epidemiological survey (2013–2014) conducted in the city of Florianópolis. A total of 1,197 older adults (778 women) over 60 years old participated in the study. Cognition, the dependent variable, was measured by the Mini-Mental State Examination (MMSE). The independent variables were the following indicators of unhealthy dietary habits: low intake of fruits and vegetables (≤ 4 servings/day); fish (< 1 serving/week); and habitual fatty meat intake (yes/no). Adjustments were made for age, education level, income, smoking status, alcohol intake, leisure-time physical activity, depression symptoms, chronic diseases, and body mass index. Simple and multiple linear regression analyses were performed, considering sampling weights and stratification by gender.

**Results:**

The mean MMSE scores for men and women were 25.15 ± 5.56 and 24.26 ± 5.68, respectively (p = 0.009). After adjustments, in women low fruit and vegetable intake (≤ 4 servings/day) was independently associated with the lowest MMSE scores. No associations were found in men. Additionally, women’s mean MMSE scores increased as their daily frequency of fruit and vegetable intake increased (p = 0.001).

**Conclusion:**

Women with low fruit and vegetable intake according to the *World Health Organization* (WHO) have lower cognition scores. Regular intake of fruits, vegetables, and fish in exchange of fatty meats may be a viable public policy strategy to preserve cognition in aging.

## Introduction

In Brazil the aging population is rapidly expanding driven by increased longevity.[1] Events associated with cognition are among the challenges of this development since even healthy aging is associated with loss of cognitive capacity stemming from functional and structural changes.[2] Loss of cognitive capacity is of concern because it impacts the independence, autonomy, and quality of life of these individuals.[3,4] Additionally, they can increase health care demands and costs[5] in Brazil’s welfare system and social assistance programs.[6] Thus, monitoring and understanding cognition determinants are critical because they can guide public policies that help to preserve cognitive capacity.

Age is the main determinant of cognition.[4,7] However, gender[8,9,10,11] and exposure to different environmental factors, such as lower education levels and income, physical inactivity, depression symptoms, and lifelong dietary habits, may also have important implications.[8,12] Regular intake of fruits, vegetables, and fish has been reported to be protective for cognition.[13,14] The positive effect is attributed to the presence of nutrients, antioxidants, and bioactive compounds like vitamins E and C, carotenoids, and flavonoids in fruits and vegetables[15,16] and polyunsaturated fatty acids (ω3) in fish.[17] In contrast, fatty meats, sources of saturated fatty acids, have a negative impact on cognitive health.[18]

In 2003 a joint WHO/FAO[19] expert consultation group presented dietary recommendations for preventing chronic diseases. The recommendations include the consumption of five or more servings of fruits and vegetables per day (400-500g/day) and limited intake of saturated fats. The failure to meet the recommended intakes of fruit, vegetables, and fish and to avoid red meat with visible fat and chicken skin are indicators of unhealthy eating habits[20]. According to the *Brazilian Surveillance of Risk and Protective Factors for Chronic Diseases by Telephone Survey* (Portuguese acronym, VIGITEL)[20], men and women have different eating habits.

In Brazil only two population-based studies[21,22] have investigated the association between fruit and vegetable intake and cognition in older adults. However, the studies were conducted in the Brazilian Southeast region and reflect the conditions of its population. Since Brazil’s territorial dimensions and cultural, social, and economic diversities promote different eating habits,[23] this study aimed to assess the association between unhealthy dietary habits and cognition in older adults from Southern Brazil.

## Methods

### Study design

This cross-sectional study analyzed data from the household- and population-based epidemiological survey, *“Condições de saúde da população idosa do município de Florianópolis/SC*: *estudo de base populacional–EpiFloripa Idoso*,*"* (Health conditions of older adults from the municipality of Florianópolis/SC: population-based study–*EpiFloripa Elderly*). The survey is a closed cohort of older adults conducted at four-year intervals. The first wave occurred in 2009–2010 and the second, in 2013–2014. The present study used data from the second wave.

The study was conducted in Florianópolis, capital of the state of Santa Catarina. Florianopolis is a coastal city located in the Brazilian South region. According to the United Nations Development Program (UNDP),[24] Florianópolis is the Brazilian state capital with the highest Human Development Index (HDI = 0.847), and the state of Santa Catarina ranks third (HDI = 0.774).

### Population and sample

The study population consisted of male and female community dwellers aged 60 years or more living in a municipal urban area.

The first wave of the survey considered the following parameters: total population of 44,460 adults, 50% prevalence of unknown outcome, sampling error of 4%, design effect of two, and confidence interval of 95% (95%CI). The study included an extra 20% to compensate for losses and 15% to control for confounders. Hence, the calculated sample size of 1,599 individuals was increased to 1,911, and 1,705 individuals were effectively interviewed (response rate of 89.2%).[25]

For the second wave of the survey, 1,485 participants of the first edition were considered eligible. Eligibility considered 217 deaths, two older adult duplications, and one older adult with incompatible age in the 2009/2010 database. A total of 129 individuals refused to participate and 159 were lost; of these, 111 were not located, totaling 1,197 interviewees (response rate of 80.6%).

The *EpiFloripa Elderly* survey was approved by the Research Ethics Committee of the Federal University of Santa Catarina under protocol number 329,650, issued on July 08, 2013 (CAAE: 16731313.0.0000.0121). All participants provided written informed consent.

### Cognition–dependent variable

Cognition was measured by the *Mini-Mental State Examination* (MMSE) validated for older Brazilians.[26] The instrument’s score ranges from 0 (least cognition) to 30 (most cognition) points. The results were presented as the total score.

### Independent variables

The following were considered unhealthy eating habits: low intake of fruits, vegetables, and fish; and intake of red meat with fat and chicken skin. The questions were taken from the national survey VIGITEL.[20] The questions have good reproducibility[27] and reflect the true frequency of consumption of specific foods. However, they do not allow the quantitative assessment of food intake.

Habitual fruit and vegetable intake was investigated by the following questions: “On a regular day, how many times do you eat fruits?”; “On a regular day, how many times do you eat cooked vegetables?” The possible answers were: once a day; twice a day; and ≥ 3 times a day for fruits; and once a day (lunch or supper); and twice a day (lunch or supper) for vegetables. The fruit and vegetable intake frequencies were added and categorized as follows: ≤ 4 times a day (inadequate) and ≥ 5 times a day, as recommended by the WHO.[19]

Fish intake frequency was investigated by the following question: “On how many days a week do you eat fish (salmon, tuna, sardine, bluefish, trout, croaker, spotted sorubim, or trahira)?” An intake < 1 once a week was considered inadequate as recommended by the WHO.[19]

Habitual intake of red meat with fat and chicken skin was investigated by the following questions: “When you eat red meat (beef, pork, lamb, or kid) with fat, do you eat the fat?” (no, I remove excess visible fat or I do not eat red meat with much fat; yes, I eat the fat); “When you eat chicken with skin, do you eat the skin?” (no, I always remove the skin or I do not eat pieces of chicken with skin; yes, I eat the skin or I eat chicken with skin).

### Adjustment variables

Demographic and socioeconomic variables consisted of age, education level (in years of formal education), and family income per member in *reais* stratified by quartiles (1st quartile: ≤ US$ 304.34; 2nd quartile: US$ 304.35 to US$ 524.45; 3rd quartile: US$ 524.46 to US$ 1,152.00 or 4th quartile: ≥ US$ 1,152. 01), considering the dollar exchange rate on November 21, 2013 (US$ 2.30).

Lifestyle determinants included smoking status (smoker; ex-smoker + never smoked: nonsmoker); alcohol intake according to the *Alcohol Use Disorders Identification Test* (AUDIT)[28](moderate use + excessive use: alcohol user; alcohol non-user); and level of physical activity determined by the *International Physical Activity Questionnaire* (IPAQ):[29]< 150 minutes/week: insufficiently active; ≥ 150 minutes/week: physically active.

Health variables consisted of self-reported medical diagnosis of hypertension (HT), diabetes mellitus (DM), cardiovascular disease (CVD), and stroke (no; yes). Depression symptoms were verified by the *Geriatric Depression Scale*–Short Form (GDS-SF) with the following cut-off points: five/six (asymptomatic/symptomatic).[30] Nutritional status was classified according to *Body Mass Index* (BMI). Body weight and height were measured as recommended by Frisancho.[31] Body weight was measured by a portable digital scale with accuracy of 100 grams. Height was measured by a portable stadiometer with accuracy of 1 mm. The cut-off points were: BMI < 22 Kg/m^2^ underweight; 22 ≤ BMI < 27 Kg/m^2^ normal weight; BMI ≥ 27 Kg/m^2^ overweight as recommended by the *Sistema de Vigilância Alimentar e Nutricional* (SISVAN, Food and Nutrition Surveillance System).[32]

### Statistical procedures

For the descriptive analysis, the variables were presented as means, standard deviations, frequencies, and proportions. The Student’s t-test and chi-square test verified gender-related differences.

The MMSE means and confidence intervals were calculated for the daily frequency of fruit and vegetable intake, using univariate analysis of the general linear model stratified by gender. The analyses were adjusted for age, education level, household income per member, leisure-time physical activity, smoking status, alcohol intake, HT, DM, CVD, stroke, depression symptoms, and body mass index. The graphs were constructed by the software Microsoft Excel (2003).

The association between unhealthy eating pattern indicators and cognition was analyzed by simple and multiple linear regression, with the results presented as regression coefficient (β) and their respective 95%CI stratified by gender. Three multiple regression models were constructed for each indicator. Model 1: adjustment for age, education level, and household income per member; Model 2: adjustment for age, education level, household income per capita, leisure-time physical activity, smoking status, and alcohol intake; Model 3: adjustment for age, education level, household income per member, leisure-time physical activity, smoking status, alcohol intake, HT, DM, CVD, stroke, symptoms of depression, and body mass index. The variables age and education level entered the models as continuous and the others, as categorical.

The analyses considered the sampling weights and were performed using the complete sample resource of the software SPSS^®^ 17.0. The significance level was set at 5% (p ≤ 0.05) and the confidence interval at 95%.

## Results

The study sample consisted of 1,197 older adults, of whom 778 (64.99%) were women. The mean ages of the women and men were 74.32 ± 7.35 years and 73.24 ± 7.15 years, respectively (p<0.001). Men had higher mean education level in years (9.74 ± 6.53) than women (7.38 ± 5.20), a significant difference (p<0.001). The mean MMSE score of 98.9% (n = 1,185) of the sample was 24.57 ± 5.65 (range 0–30) because 12 individuals were not assessed. Men had higher mean MMSE score (25.15 ± 5.56), than women (24.26 ± 5.68) (p = 0.009).

Table 1 shows the distribution of the participants according to the study variables and gender. Men had higher frequencies of poor habits than women, such as intake of meat with fat, alcohol intake, and tobacco use.

**Table 1:** Sample characteristics stratified by gender. *EpiFloripa Elderly*, 2013–2014.

Variables	All		Men		Women		P
	N	%	N	%	N	%	
**Fruit and vegetable intake**							
≥ 5 times/day	381	32.2	125	34.6	256	65.4	0.450
≤ 4 times/day	799	67.8	285	37.6	514	62.4	
**Fish intake**							
≥ Once/week	632	51.1	228	38	404	62	0.518
< Once/week	563	48.9	189	35.8	374	64.2	
**Intake of red meat with fat**							
No	930	87	299	34.7	631	65.3	**< 0.001**
Yes	148	13	94	65.8	54	34.2	
**Chicken skin intake**							
No	982	89.7	311	33.9	671	66.1	**< 0.001**
Yes	122	10.3	67	59.5	55	40.5	
**Household income per member**							
4th quartile (highest)	298	24.9	126	45.4	172	54.6	**< 0.001**
3rd quartile	299	25.0	92	32.4	207	67.6	
2nd quartile	273	22.8	105	40.4	168	59.6	
1st quartile	325	27.2	93	29.2	292	70.8	
**Smoking status**							
Non-smoker	1112	92.8	375	35.6	737	64.4	**0.006**
Smoker	83	7.1	42	53.9	41	46.1	
**Alcohol intake**							
No	750	62.1	182	24.6	568	75.4	**< 0.001**
Yes	446	37.9	235	57	211	43	
**Leisure-time physical activity**							
Adequately active	608	50.8	252	62.3	356	46	**< 0.001**
Inadequately active	589	49.2	167	37.7	422	54	
**Hypertension**							
No	415	34.9	183	48.5	232	51.5	**< 0.001**
Yes	780	65.1	234	30.7	546	69.3	
**Stroke**							
No	1061	90.2	361	36	700	64	**0.040**
Yes	135	9.8	56	45.3	79	54.7	
**CVD**							
No	805	67.8	276	35.3	529	64.7	0.154
Yes	391	32.2	141	40.2	250	59.8	
**DM**							
No	895	76.3	331	39.4	564	60.6	**0.014**
Yes	301	23.7	86	28.9	215	71.1	
**Depression symptoms**							
No	906	80.9	334	38.7	572	61.3	0.055
Yes	223	19.1	62	30.6	161	69.4	
**BMI**							
Normal weight	417	36.2	170	44.4	247	55.6	**0.014**
Underweight	117	10.1	37	30.8	80	69.2	
Overweight	596	53.7	189	33.8	407	66.2	

Complex sample; Chi-square test. Significance level of 5% (p ≤ 0.05). CVD = cardiovascular disease. DM = diabetes mellitus.BMI = body mass index.

Women had significantly higher frequencies of HT, DM, stroke, depression symptoms, overweight, underweight, and physical activity insufficiency than men.

Tables 2 and 3 show the results of the association between the indicators of unhealthy eating habits and cognition in women and men, respectively.

**Table 2:** Relationship between indicators of unhealthy food habits (inadequate food intake) and cognition in women. *EpiFloripa Elderly*, 2013/2014.

Inadequate intake:	*Crude analysis*			*Model 1*			*Model 2*			*Model 3(final)*		
	Β	95%CI	P	Β	95%CI	P	Β	95%CI	P	β	95%CI	p
**Fruits and vegetables**	-1.934	-2.939; -0.929	**<0.001**	-1.148	-1.987; -0.309	**0.008**	-1.085	-1.980; -0.191	**0.018**	-1.004	-1.376; -0.631	**<0.001**
**Fish**	-1.107	-2.014; -0.199	**0.018**	-0.448	-1.172; 0.275	0.221	-0.255	-0.918; 0.409	0.448	0.073	-0.312; 0.458	0.708
**Red meat with fat**	0.702	-1.026; 2.429	0.421	-0.031	-1.656; 1.594	0.970	-0.167	-1.824; 1.490	0.841	0.171	-0.873; 1.216	0.745
**Chicken skin**	1.248	-0.034; 2.530	0.056	1.350	-0.042; 2.743	0.057	1.047	-0.290; 2.384	0.123	0.290	-0.756; 1.336	0.583

Complex sample, simple and multiple linear regression. Significance level of 5% (p ≤ 0.05).Inadequate intake defined as: Fruits and vegetables (≤ 4 servings/day); Fish (< once/week); Chicken skin (yes); Red meat with fat (yes).Crude analysis. Model 1: Crude analysis, age, education level, and household income per member. Model 2: Crude analysis, age, education level, and household income per member, smoking status, alcohol intake, and leisure-time physical activity. Model 3 (final): Crude analysis, age, education level, and household income per member, smoking status, alcohol intake, leisure-time physical activity, DM, HT, CVD, stroke, depression symptoms, and BMI.

**Table 3:** Relationship between indicators of unhealthy food habits (inadequate food intake) and cognition in men. *EpiFloripa Elderly*, 2013/2014.

Inadequate intake:	*Crude analysis*			*Model 1*			*Model 2*			*Model 3(final)*		
	β	95%CI	P	Β	95%CI	P	Β	95%CI	P	β	95%CI	p
**Fruits and vegetables**	0.639	-1.096; 2.374	0.446	1.449	0.019; 2.880	**0.047**	1.545	0.071; 3.020	**0.040**	0.298	-0.393; 0.968	0.380
**Fish**	-1.078	-2.398; 0.243	0.108	-0.407	-1.568; 0.755	0.488	-0.350	-1.501; 0.801	0.547	-0.441	-1.059; 0.177	0.160
**Red meat with fat**	0.149	-1.303; 1.601	0.839	0.642	-0.675; 1.959	0.335	0.699	-0.609; 2.006	0.291	0.505	-0.415; 1.424	0.278
**Chicken skin**	-0.692	-2.814; 1.430	0.518	0.228	-1.263; 1.719	0.762	0.078	-1.245; 1.401	0.907	0.008	-1.103; 1.120	0.988

Complex sample, simple and multiple linear regression. Significance level of 5% (p ≤ 0.05). Inadequate intake defined as: Fruits and vegetables (≤ 4 servings/day); Fish (< once/week); Chicken skin (yes); Red meat with fat (yes). Crude analysis. Model 1: Crude analysis, age, education level, and household income per member. Model 2: Crude analysis, age, education level, and household income per member, smoking status, alcohol intake, and leisure-time physical activity. Model 3(final): Crude analysis, age, education level, and household income per member, smoking status, alcohol intake, leisure-time physical activity, DM, HT, CVD, stroke, depression symptoms, and BMI.

In women crude analysis of fruit and vegetable intake (≤ 4 times/day) and fish (< once a week) were associated with the lowest MMSE scores. After adjustments, the variable fruit and vegetable intake (≤ 4 servings/day) remained associated with lower MMSE scores. In the final model, low intake of fruits and vegetables reduced the MMSE score by -1.004 (-1.376; -0.631) points (Table 2).

Although an association was found between fruit and vegetable intake (≤ 4 times/day) and MMSE score in Models 1 and 2, the association disappeared in the final model (Table 3).

**Fig 1:**
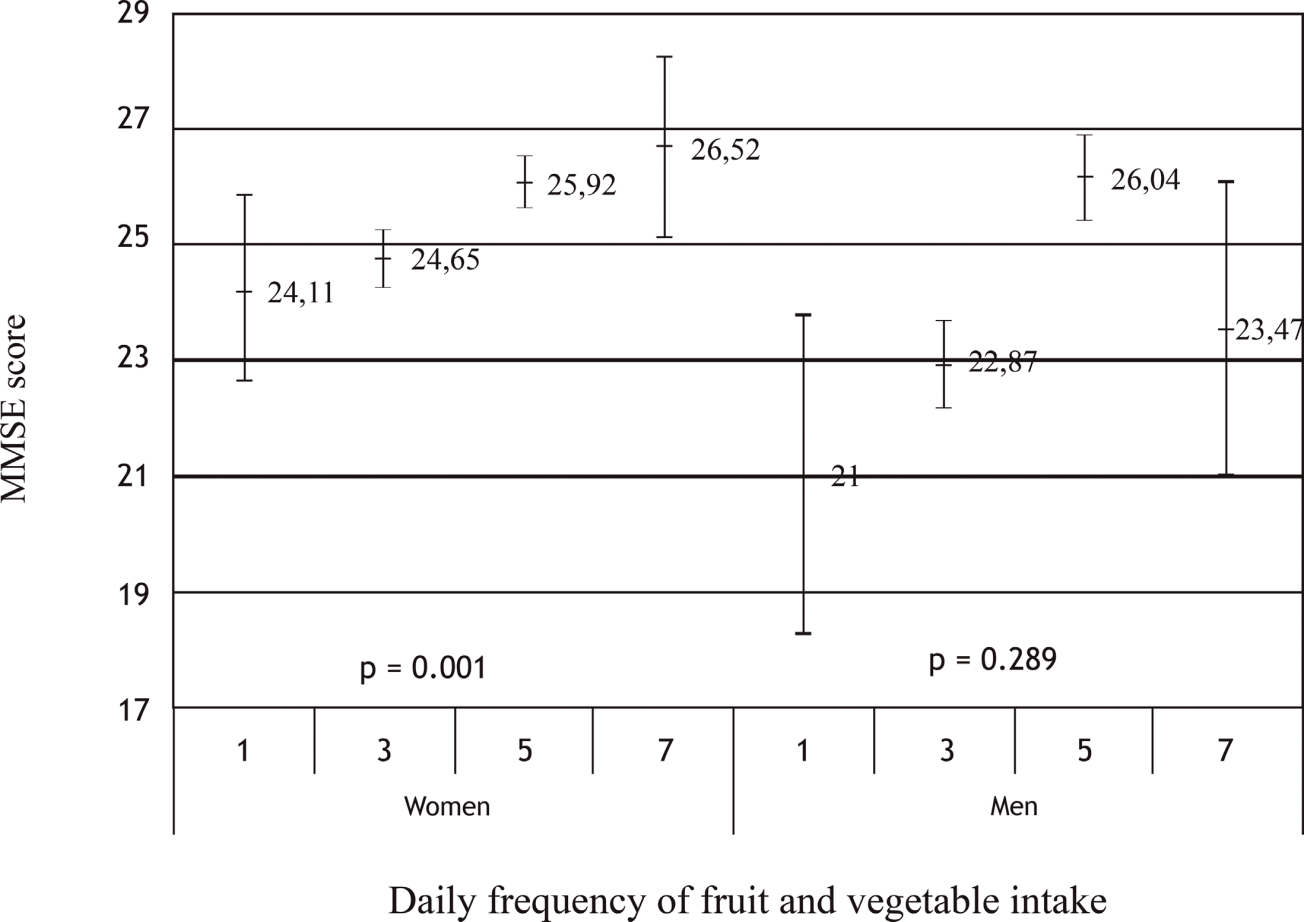
Univariate analysis of MMSE means and confidence intervals according to the daily frequency of fruit and vegetable intake for both genders.

Univariate analysis showed positive interaction between MMSE score and the daily frequency of fruit and vegetable intake in women (p = 0.001) (Fig 1).

## Discussion

The results showed that although men have higher MMSE scores, the independent association between inadequate fruit and vegetable intake (≤ 4 times/day) and lower MMSE scores occurred only in women. Additionally, the daily frequency of fruit and vegetable intake presented a positive interaction with women’s MMSE score.

Higher cognition scores in men are consistent with other national[8,9] and foreign[10,11] studies. However, other studies found lower MMSE scores in men.[21,33]

The study analyses were stratified by sex as men and women have specific biological characteristics that may partly explain differences in cognition.[34] Factors such as age, social determinants such as education level and income,[4,8] physical inactivity, depression symptoms,[35] chronic diseases,[36] smoking status, alcohol intake, and genetic factors[8,37] also explain the variation in cognition, so their combined effect may be greater than in isolation.

The study showed that men had a higher frequency of poor habits and lower frequencies of diseases, overweight, underweight, depression symptoms, and mean age. They also had higher education level and income, which may have contributed to their MMSE score.

There was a high prevalence of inadequate fruit and vegetable intake according to the WHO recommendations[19] regardless of gender. These results corroborate with those of the *Brazilian Family Budget Survey* (Portuguese acronym POF) 2008–2009. POF found that less than 10% of the general population had adequate fruit, vegetable, and legume intakes according to the WHO[19] and Brazilian Food Guide.[23]

In women inadequate fruit and vegetable intake was associated with lower cognitive scores. This association persisted after adjustment for the control variables. Additionally, the MMSE score presented a significant positive interaction with the daily frequency of fruit and vegetable intake in women. These results have not been found for men. The higher mean values of MMSE for men compared with women may explain the lack of association for them.

A Brazilian study of 1,558 older adults from Minas Gerais state found that fruit and vegetable intake below five servings a week was associated with lower MMSE scores (OR = 1.94; 95%CI 1.46 to 2.59).[21] Moreover, a study conducted in São Paulo found that fruit and vegetable intake (≥ 5 servings/day) was associated with a smaller prevalence of compromised cognition in older adults from lower-income areas (OR = 0.53; 95% CI 0.31 to 0.89).[22] Concordantly, a Chinese study found that adequate fruit and vegetable intake was associated with lower risk of compromised cognition in women (OR = 0.73; 95% CI 0.54 to 1.00).[38] In studies of Chen et al[33]. and Wang et al[39] lower intakes of vegetables and/or legumes were associated with cognitive impairment, and regular fruit intake was not associated with outcome. Possible explanations for these apparently different results is supported by the fact that vegetables seem to have a higher amounts of vitamin E. Leafy green and vegetables are also usually consumed with added fats, which increase the absorption of vitamin E, and carotenoids and flavonoids[15].

The most accepted hypothesis to explain the protective effect of fruits and vegetables is the combination of fibers, antioxidant components, vitamins C and E, carotenoids, and other bioactive components, like flavonoids[16,40,41]. The potential effect of antioxidants involves the suppression of inflammation and injury of neuronal cells, and the promotion of cognition[42].

The study results indicate the need of compliance with the recommended fruit and vegetable intake, even in this group, living in an economically favorable area. It is possible that the consumption of fruits and vegetables (≥ 5 times daily) may have protective effects on cognition of older adults living in economically vulnerable areas.

A fish intake estimate of < once a week can be considered high in this population. Florianopolis is an island, so availability and access to fish are favored by its location and local fishing culture. In women, only simple analysis found an association between fish intake < once a week and MMSE score.

Similar to this finding, the prospective study of 3,294 older French, *“Supplementation with Antioxidant Vitamins and Minerals Study”* (SUVIMAX),[43] did not find an association between higher fish intake and global cognitive function measured by the MMSE. On the other hand, cognitive impairment was less frequent in older adults who consumed fish regularly in the 13 years prior to the assessment (OR = 0.72; 95%CI 0.56 to 0.92). Additionally, the *“Cardiovascular Health Study—Cognition Study”* (CHS-CS) found that older adults who consumed fish four or more times a week had higher MMSE scores.[44]

The frequency of older adults who reported regularly eating red meat with visible fat or chicken without removing the skin was similar and higher in men than in women. A similar result was found by the VIGITEL study, conducted in Brazilian state capitals and the Federal District: roughly 20% of individuals aged 65 years or more ate fatty meats regularly; intake frequency was higher in men (28.0%) than in women (13.9%).[45] The different intakes by gender may be related to their social roles, where women have higher nutrition knowledge than men.[46] The present study did not find associations between the habitual intake of fatty meats and MMSE for either gender.

Although the study results did not find an association between the intake of fish and/or fatty meats and cognition, a diet high in fish and low in fatty meats is recommended for older Brazilians[47] and by the WHO’s report on diet, nutrition, and the prevention of chronic diseases.[19] Fish is a source of linolenic polyunsaturated fatty acids,[48] so regular intake may protect cognition in old age.[14,17,49,50] Inversely, regular fatty meat intake has been pointed out as a risk factor for cognitive health due to the presence of saturated fatty acids.[14,47] The lack of association between fish and cognition, as well as between fatty meats and cognition, may be due to the absence of quantitative food intake assessment.

This study has limitations and strengths. One limitation is the instrument. Although the MMSE is a widely used screening tool in epidemiological studies, it is influenced by educational level. In Brazil different cut-off points are proposed for individuals with low education levels,[7,51,52,53] resulting in conflicting results when administered to the same population. Therefore, in the absence of consensus of an optimal cut-off point, this study considered the MMSE a continuous variable, which may also be a limitation.

Another limitation is the use of simple questions for assessing food intake frequency. These are used annually by the nationwide VIGITEL study, which only investigates current food intake frequency and some protective factors for chronic diseases. Although it has good reproducibility; it does not allow the quantitative assessment of food intake or adjustment for total caloric intake. Future studies should assess eating habits using a quantitative approach. A final limitation is the specification of fish species (salmon, tuna, sardine, bluefish, trout, croaker, spotted sorubim, or trahira), excluding the consumption of other species, such as mullet, available in Florianópolis.

The strengths are the representativeness of the sample and the methodological cogency in all stages of the *EpiFloripa Elderly* study. This study provides the first estimates of the association between food intake indicators and cognition in older community dwellers from Florianópolis. Thus, the results may contribute to the understanding of cognition-related factors and to the promotion of public health strategies for older adults.

Given the socioeconomic reality of Florianópolis, which has high HDI[24], we suggest caution when extrapolating the results to older adults from other Brazilian urban centers and other middle-income countries. The cultural, social, and economic diversities of different populations distinctively determine education level, occupation, income, and health behaviors, all which impact cognition. Hence, the study results reflect the particularities of older adults from Florianópolis.

## Conclusion

This study shows that women who did not have adequate fruit and vegetable intake according to the WHO had lower MMSE scores. In men lower mean age and higher education levels and income provide evidence that biological and social factors are strong determinants of cognition.

The results indicate a need to prioritize public health policies in Florianópolis that value healthy lifestyles in order to preserve cognitive capacity.

Recognizing the Brazilian financial and structural limitations, simple and inexpensive actions that encourage fruit, vegetable, and fish intake in place of fatty meats should be presented to the Brazilian population to improve cognition.
